# Alisklamp versus Conventional Dorsal Slit Circumcision: A Multicentric Randomized Controlled Trial

**DOI:** 10.3390/jcm13154568

**Published:** 2024-08-05

**Authors:** Mustafa Azizoglu, Toni Risteski, Sergey Klyuev

**Affiliations:** 1 Department of Pediatric Surgery, Istanbul Esenyurt Hospital, 34510 Istanbul, Turkey; 2 Department of Stem Cell and Tissue Engineering, Health Science Institute, Istinye University Medical School, 34510 Istanbul, Turkey; 3 Department of Pediatric Surgery, Medical Faculty, Ss. Cyril and Methodius University of Skopje, 1020 Skopje, North Macedonia; drtonirist@yahoo.com; 4 Department of Pediatric Surgery, AO GK MEDSI, 102151 Moscow, Russia; proklyuev@gmail.com

**Keywords:** Alisklamp, male circumcision, dorsal slit technique, sutureless, children

## Abstract

**Background:** There are numerous methods of circumcision performed worldwide, typically classified into two main groups: conventional surgical techniques and various device-assisted techniques. Each method has its own advantages, limitations, and potential complications. The aim of this study was to compare outcomes of the Alisklamp technique versus the dorsal slit technique in male circumcision procedures. **Method:** This multicenter RCT compared the dorsal slit and Alisklamp techniques for circumcision, assessing patient demographics and intraoperative and postoperative outcomes. All patients, under local anesthesia via dorsal penile nerve block, were discharged on the same day and followed up at 24–48 h, 1 week, and 1 month. **Results:** A total of 180 patients enrolled, and 166 patients were included. The study compared postoperative outcomes between the Alisklamp (AK) and dorsal slit (DS) circumcision techniques in 166 patients. Key findings included significantly higher penile edema in the DS group (19%) compared to the AK group (2.4%) (*p* < 0.001), with severe edema occurring only in the DS group. Wound gaping was more common in the AK group (8.3%) compared to the DS group (1.2%) (*p* = 0.030). Skin tunnels were observed only in the DS group (9.5%) (*p* = 0.004). There were no significant differences in nausea, vomiting, bleeding, necrosis, infection, wound dehiscence, chordee, rotational anomalies, or secondary phimosis between the groups. Mean operation time was lower in the AK group than the DS group (7.8 min vs. 15.5 min; *p* < 0.001). **Conclusions:** The Alisklamp technique is recommended as the preferred method for circumcision because it minimizes complications, shortens the procedure time, and is easy to apply.

## 1. Introduction

Male circumcision, the surgical removal of the foreskin from the penis, has been practiced for thousands of years, primarily for religious, cultural, or medical reasons [1,2]. Over time, the surgical techniques employed for circumcision have evolved, aiming to minimize pain, reduce complications, enhance cosmetic outcomes, and optimize recovery times [1,2,3].

There are numerous methods of circumcision performed worldwide, generally classified into two main groups: conventional surgical techniques and multiple device-assisted techniques [4,5]. Each technique has its own advantages, limitations, and complications [6]. The dorsal slit technique is a traditional procedure with a long-standing history. The technique involves making a slit on the dorsal side of the foreskin, which allows the foreskin to be peeled back and excised, much like removing a sleeve [1,7,8]. The approach provides good exposure of the penile glans and allows for precise removal of the foreskin. As with all surgical methods, it comes with its own set of advantages and challenges [1,8,9].

In addition to the traditional surgical technique, many devices have entered the medical supply market, facilitating the process and improving the outcome of circumcision [4,10,11]. About 20 devices are available for infant and adult male circumcision [10,11,12,13,14,15,16]. The protection and effectiveness of device-assisted male circumcision are equivalent to or greater than traditional surgery [15,17]. These devices have the potential to simplify the process and improve acceptability among patients.

One of the devices designed to assist circumcision is the Alisklamp [15,17]. The Alisklamp technique is a more recent innovation designed to provide a bloodless circumcision experience. This technique employs a specially designed clamp to achieve hemostasis while the foreskin is excised [15].

The aim of this study was to compare outcomes of the Alisklamp technique and the dorsal slit technique in male circumcision procedures.

## 2. Materials and Methods

### 2.1. Patients and Design

This research was designed as a multicentric randomized controlled trial (RCT) to compare the efficacy and outcomes of the dorsal slit technique and the Alisklamp (Ankara, Turkey) technique in circumcisions. The study was conducted at Necmi Kadioglu Hospital and University clinic for pediatric surgery within the medical faculty of Ss Cyril and Methodiusi between 20 December 2023 and 1 March 2024. This study was approved by the University clinic for pediatric surgery of Ss Cyril and Methodiusi Skopje, North Macedonia (approval number: 0302/447) and registered at www.clinicaltrials.gov (NCT06177834) (access on 1 April 2024). Informed consent was obtained from parents.

**Figure 1:**
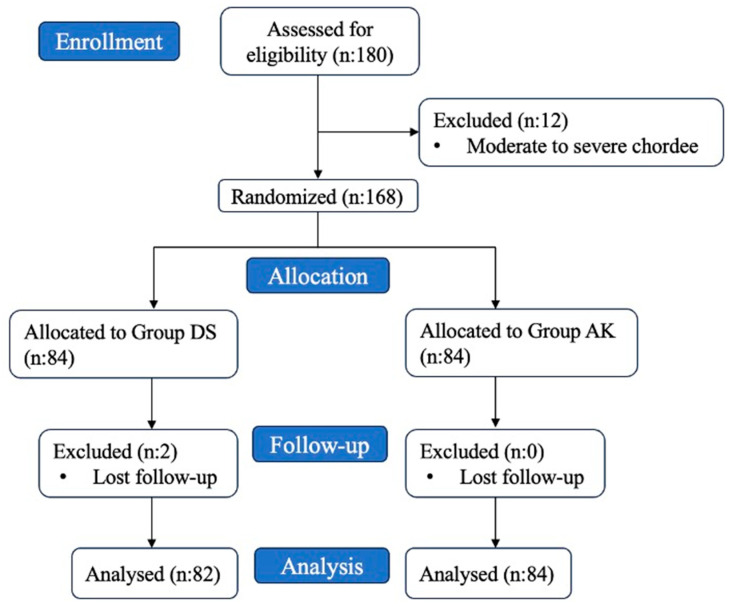
Flow chart.

A total of 180 patients were randomly assigned to either the dorsal slit technique (DS) group or the Alisklamp (AK) technique group using computer-generated random numbers. The process is summarized in a flow chart in Figure 1.

### 2.2. Inclusion Criteria

- Male patients aged 0–2 years or 6–18 years.

### 2.3. Exclusion Criteria

- Male patients aged 2–6 years (psychosexual concern [18]).
- Penile chordee, hypospadias, or previous penile surgery.
- Congenital penile anomalies (such as hypospadias, penoscrotal web, penile rotation > 30°, or micropenis).
- Local infection or significant diaper rash.
- A history of hereditary bleeding disorders.
- Parental refusal of circumcision method randomization.
- Previous circumcision.
- Comorbid disease.

The study evaluated patient demographic data, intraoperative findings (operation time, and complications), early postoperative outcomes (nausea, vomiting, bleeding, penil oedema, pain score, analgesic needs, necrosis, and infections), and late postoperative outcomes (wound dehiscence, skin tunnel, chordee, and rotational anomalies). All patients were circumcised under local anesthesia. Dorsal penile nerve block technique was applied for local anesthesia in all patients. All patients were discharged on the same day. Patients were assessed at least three times following circumcision: 24–48 h, 1 week, and 1 month after the procedure.

### 2.4. Dorsal Penil Nerve Block

The procedure was carried out in a supine position. Following skin preparation with povidone–iodine and palpation of the lower border of the symphysis pubis, the base of the penis was carefully retracted downward. A 30 G needle was inserted into the midline at a 75° angle to the skin. The needle was then partially withdrawn and redirected towards the right side, where half of the local anesthetic (LA) was administered after making bone contact with the symphysis pubis. The same procedure was then performed on the left side [19].

### 2.5. Postoperative Pain Assessment

Children’s pain levels were assessed using the Face, Legs, Activity, Crying, Consolability Scale (FLACC). The total score was measured on a scale from 0 to 10, where 0 indicates comfort, 1–3 indicates mild discomfort, 4–6 indicates moderate pain, and 7–10 indicates severe discomfort or pain. If the FLACC score was greater than 4, 10–15 mg/kg of paracetamol was planned to be administered orally as rescue analgesia [20,21] (Table 1).

### 2.6. Surgical Procedures

During the dorsal slit procedure, after local anesthesia, the prepuce was held bilaterally and from the posterior with three mosquito clamps. The surgeon then excised the prepuce skin using scissors, controlled any bleeding, and joined the mucosa and skin using 5/0 rapid Vicryl sutures. After the circumcision, the wound was wrapped in a gauze bandage, and parents were instructed on potential issues that might arise at home, including managing the dressing, dealing with bleeding, recognizing signs of infection, and when to seek emergency medical attention. The dressing was removed in all patients 24–48 h postoperatively [8,9].

**Figure 2:**
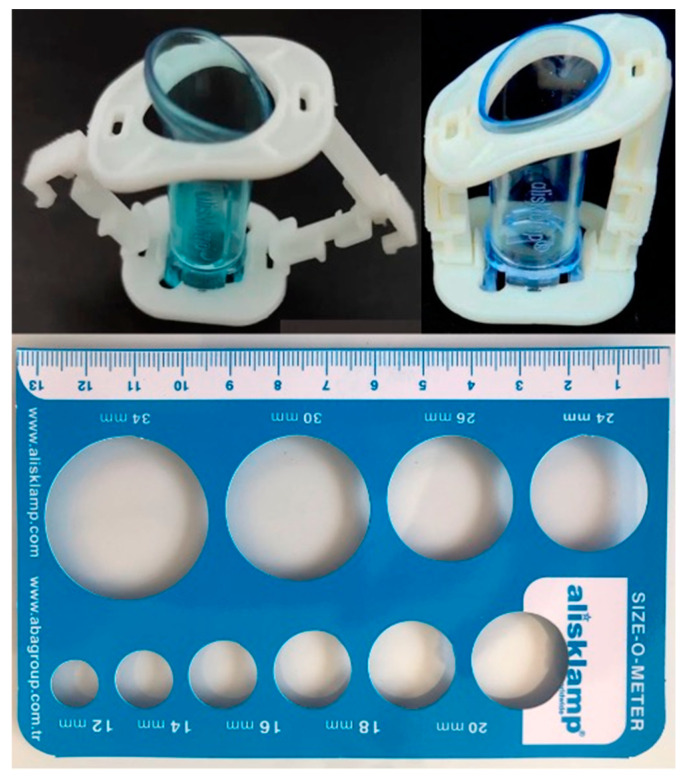
AKD kit.

**Figure 3:**
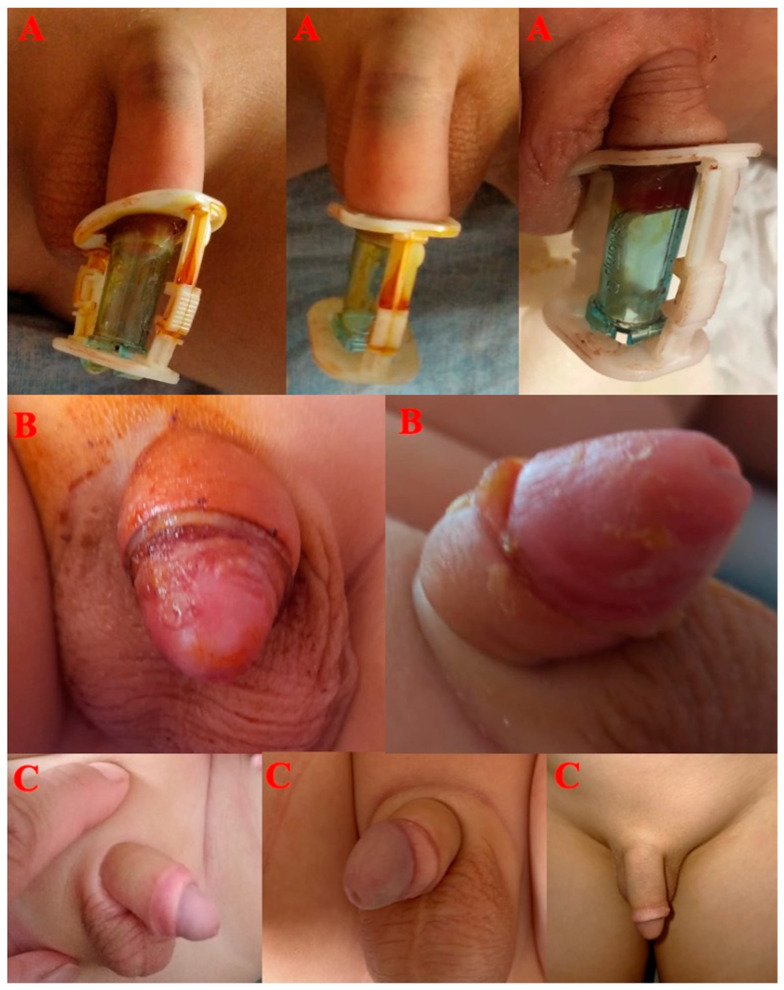
(**A**). Placement of AK; (**B**). At 1 week after removal AK; (**C**): At 1 month after removal AK.

Prior to applying the Alisklamp device, the size of the penis was determined using a template from the Alisklamp device (AKD) kit (Figure 2). The process begins by fitting a device tube over the prepuce, ensuring the preputium is pulled up over the tube. The lengths of the mucosa and skin to be cut were then adjusted accordingly, and the clamp was secured by closing the upper and lower arms of the AKD. For cases involving tight phimosis, a 3–5 mm dorsal slit was made to fully separate the inner and outer preputial layers. The Alisklamp was subsequently removed by the surgeon 24–48 h after the surgery (Figure 3). Once all preputial adhesions were dissected, the coronal sulcus just behind the glans penis was completely exposed. The preputium was drawn back to its neutral position, and the 12 and 6 o’clock positions were marked for reference. The penile skin was then retracted through the pubis using finger pressure at the base of the penis, and the coronal sulcus projections were marked. These markings are essential in defining the estimated skin length of the erect penis. Following this, an inner tube was inserted inside the preputium. If the preputial neck was too narrow to allow the tube’s insertion, a dorsal slit was made to facilitate the process. With the outer chamber set in an unlocked position, the inner mucosa was tailored using an external light source for transillumination, which helps in determining the mucosal incision level. The locked arms were then set at the 6 and 12 o’clock positions. The outer chamber was positioned just above the coronal sulcus markings, and the arms were locked. An incision was carried out, leaving a 1- or 2 mm safety margin over the locked chamber. No dressing is needed in the Alisklamp technique [15,17]. Alisklamp removal is a quick procedure, taking only 5 s and requiring no local anesthesia.

After the dressing was removed in the DS group and the Alisklamp device was removed in the AK group, all patients were instructed to use a topical antibiotic ointment for one week.

### 2.7. Statistical Analysis

The data collected for our study were analyzed using Jamovi software 2.4.1. Descriptive data were presented as counts and percentages for categorical variables and as means and standard deviations for continuous variables. The Pearson Chi-square test was used to identify differences between groups for categorical variables. The Kolmogorov–Smirnov test was employed to assess the normality of the distribution for continuous variables. For comparisons between two variables, the independent t-test was used. A *p*-value of less than 0.05 was considered statistically significant.

## 3. Results

**Figure 4:**
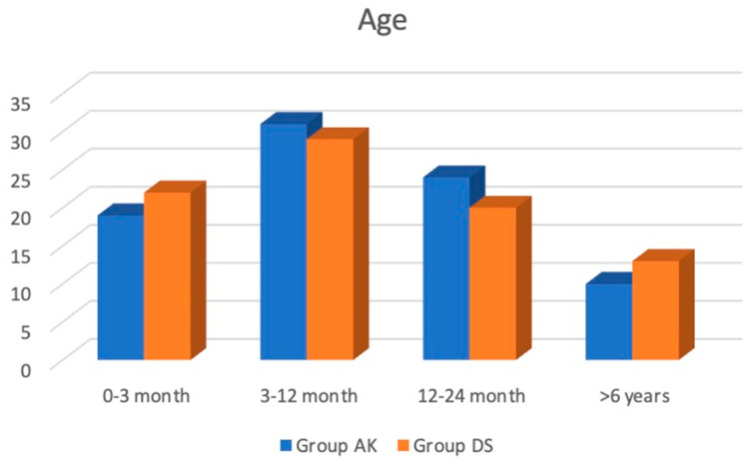
Age at operation.

A total of 180 patients were enrolled in our study. For the final analysis, 166 patients were included, with 82 in Group DS and 84 in Group AK. The participants’ ages were distributed across four categories: 0–3 months, 3–12 months, 12–24 months, and over 6 years. There was no significant difference in age distribution between the two groups (*p* = 0.791). Specifically, in Group AK, 19 (22%) were aged 0–3 months, 31 (37%) were aged 3–12 months, 24 (29%) were aged 12–24 months, and 10 (12%) were older than 6 years. In Group DS, 22 (26%) were aged 0–3 months, 29 (35%) were aged 3–12 months, 20 (24%) were aged 12–24 months, and 13 (15%) were older than 6 years (Figure 4).

The occurrence of severe rotational anomaly was observed to be 0% (n = 0) in Group AK and 1% (n = 1) in Group DS, with a *p*-value of 0.316, indicating no significant difference between the groups. Similarly, meatal stenosis was noted in 0% (n = 0) of Group AK and 1% (n = 1) of Group DS, also yielding a non-significant *p*-value of 0.316. Both groups had an equal number of preoperative urinary tract infections (UTIs), with 5% (n = 4) in each group (*p* = 1.000). Preoperative phimosis was reported in 6% (n = 5) of Group AK and 5% (n = 4) of Group DS (*p* = 0.732). When examining the reasons for circumcision, the distribution was identical between the groups. Both Group AK and Group DS reported that 5% (n = 4) of the subjects underwent circumcision due to UTIs, while the remaining 95% (n = 80 in Group AK and n = 78 in Group DS) cited religious reasons as the primary motivation (*p* = 1.000) (Table 2).

**Figure 5:**
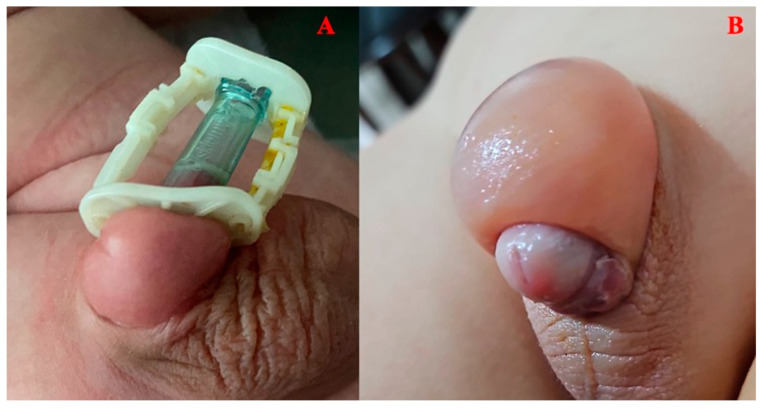
(**A**). Mild oedema in the AK group; (**B**). Severe eodema in the DS group.

Regarding postoperative complications, the incidence of nausea was 3.6% (3/84) in Group AK and 7.1% (6/82) in Group DS, which was not statistically significant (*p* = 0.304). Similarly, vomiting occurred in 2.4% (2/84) of patients in Group AK and 4.8% (4/82) in Group DS, with no significant difference (*p* = 0.406). Penile edema was significantly more common in Group DS (19%, 16/82) compared to Group AK (2.4%, 2/84) (*p* < 0.001). The degree of edema also differed significantly between the groups (*p* = 0.019). In Group AK, both cases of edema were mild (100%), while in Group DS, two cases (12.5%) were mild, seven cases (43.8%) were moderate, and seven cases (43.8%) were severe. Patients with mild and moderate oedema experienced spontaneous resolution. Among the seven patients with severe oedema, one patient, who experienced pain and difficulty urinating due to oedema, underwent bladder catheterization with a 6 Fr feeding catheter. The feeding catheter was removed after 24 h (Figure 5). 

Postoperative bleeding occurred in one patient (1.2%) in Group AK and four patients (4.8%) in Group DS, with no statistically significant difference (*p* = 0.173). In four of the five patients who experienced bleeding, the bleeding was controlled with tight local dressings. In one patient from the DS group, additional suture placement was required. There were no cases of necrosis in either group. The incidence of local infection was equal in both groups, with 1.2% (1/84) in Group AK and 1.2% (1/82) in Group DS (*p* = 1.000). The infections in both patients resolved with oral antibiotics and topical ointment. Wound dehiscence occurred in 0% (0/84) of Group AK and 1.2% (1/82) of Group DS (*p* = 0.316). After wound dehiscence, secondary healing was allowed, resulting in a complication-free recovery. This patient did not require additional surgical intervention. Wound gaping occurred in seven patients (8.3%) in group AK, compared to one patient (1.2%) in group DS (*p* < 0.030). The formation of a skin tunnel was observed in none of the patients in Group AK but in 9.5% (8/82) of the patients in Group DS (*p* < 0.004). There were no cases of chordee or rotational anomaly after the procedure in either group. No cases of secondary phimosis were observed in any group. Additionally, need for pain relief at home on more than two occasions was recorded for each group. In Group AK, 16 patients (19%) required pain relief more than twice, compared to 13 patients (15.5%) in Group DS (*p* = 0.540) (Table 3).

**Figure 6:**
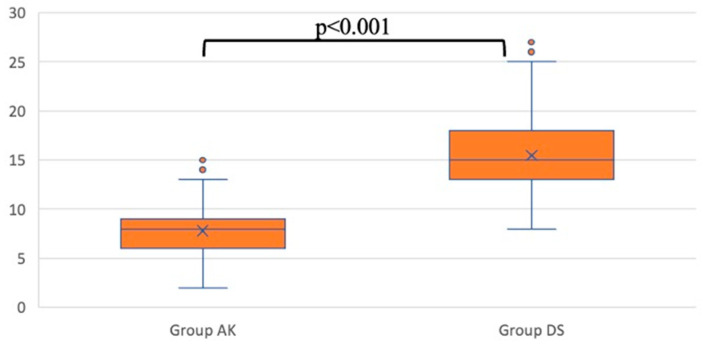
Operation time (minutes).

The operation times (skin to skin, without dressing time) for the two groups showed a significant difference. In Group AK, the average operation time was 7.8 ± 2.6 min, whereas in Group DS, the average operation time was 15.5 ± 4.5 min (*p* < 0.001) (Figure 6).

**Figure 7:**
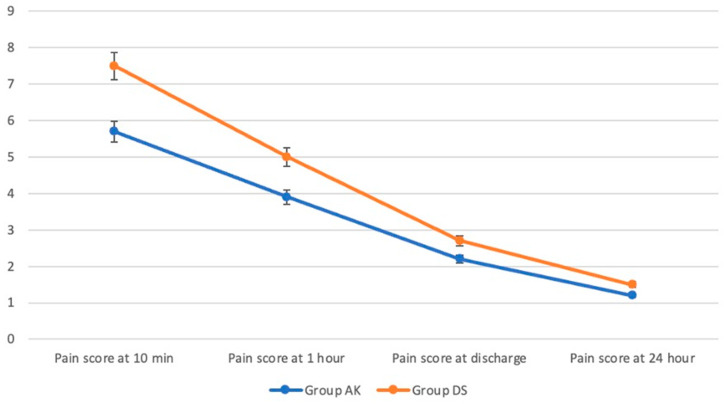
Pain scores.

The pain scores for the two groups were compared at various time points post operation. At 10 min, the pain score for Group AK was 5.7 ± 1.6, while for Group DS, it was 7.5 ± 1.9, (*p* < 0.001). At 1 h post operation, Group AK had a pain score of 3.9 ± 1.8 compared to 5 ± 1.9 in Group DS (*p* = 0.04). Upon discharge, the pain score for Group AK was 2.2 ± 0.8, whereas for Group DS it was 2.7 ± 1.2, with no significant difference (*p* = 0.129). At 24 h post operation, the pain score for Group AK was 1.2 ± 1, compared to 1.5 ± 1.2 for Group DS, also showing no significant difference (*p* = 0.235) (Figure 7).

## 4. Discussion

In this study, we found that Alisklamp technique significantly reduced operation time and penile oedema rate and degree compared to the conventional dorsal slit technique. Pain scores were lower in the Alisklamp group at 10 min and 1 h post operation. However, there was no significant difference in the use of pain relievers at home or other complications such as infections and wound dehiscence. Additionally, the incidence of skin tunnel formation was significantly lower in the Alisklamp group compared to the conventional dorsal slit group.

The Alisklamp technique, also known in the literature as the Smart Clamp Circumcision Device [22], has been demonstrated to be a safer, easier, and quicker method of circumcision [17,22,23]. This technique eliminates the need for suturing and hemostasis, thereby shortening the operation time. Furthermore, it can be performed easily without assistance or excessive tools; only one or two mosquito clamps are sufficient to pull the mucosa and foreskin over the clamp [17,23,24].

In the study conducted by Aldemir et al., the median operation time for conventional circumcision was 18 min, while for the Alisklamp technique, it was 8 min [22]. Similarly, Senel and colleagues reported an average operation time of 23 min for conventional circumcision and 4.5 min for the Alisklamp technique [24]. In our study, the average operation time was 7.8 ± 2.6 min for the Alisklamp group and 15.5 ± 4.5 min for the conventional circumcision group. As demonstrated in our study and supported by these two other studies in the literature, the Alisklamp technique reduces the operation time by at least half compared to conventional circumcision.

In the literature, infection rates for plastic clamp circumcision techniques are reported to be lower or similar compared to conventional circumcision. In our study, we observed infections in two patients: one in the DS group and one in the AK group. Most surgeons define infection as increased hyperemia and edema with pus. However, statistically higher rates of penile edema were reported with the Alisklamp in studies by Suzen et al. [17] and Karadag et al. [25]. Contrary to these findings, our study found a higher incidence of edema in the conventional circumcision group.

One concern with the Alisklamp is the potential for bleeding if it is removed too early. Therefore, it is generally recommended to remove the Alisklamp later in older patients [22]. However, in our study, the majority of patients were under 2 years old, with only a small number over 6 years old. Consequently, the Alisklamp was removed in all patients within 24–48 h. For adolescents aged 15–20, it might be advisable to remove the Alisklamp at a later time to minimize the risk of bleeding. In our study, no significant bleeding was observed in the Alisklamp group.

In the study conducted by Essa and colleagues, the percentage of patients requiring pain relief three or more times after undergoing the Alisklamp procedure was found to be 30% [15]. In our study, the rate of patients needing painkillers three or more times in the Alisklamp group was 19%. When compared to the other group, this difference was not statistically significant.

An important issue with sutured circumcision techniques is the formation of a skin tunnel [26]. On the other hand, sutureless techniques often face the problem of wound gaping. Bawazir and colleagues found that the incidence of skin tunnels was 15% in sutured circumcisions, while no skin tunnels were observed in the sutureless group. Conversely, wound gaping was observed in 1.1% of the sutured group and 8.5% of the sutureless group [26]. In our study, wound gaping occurred in seven patients (8.3%) in the Alisklamp group (Group AK) compared to one patient (1.2%) in the conventional circumcision group (Group DS), with a p-value of 0.030. Additionally, no skin tunnels were observed in Group AK, whereas 9.5% (8/82) of the patients in Group DS experienced skin tunnel formation (*p* = 0.004). A significant advantage of the Alisklamp and other sutureless methods is the absence of skin tunnel formation, leading to a more cosmetically appealing outcome.

One of the significant complications following circumcision is postoperative secondary phimosis. Süzen et al. reported a secondary phimosis rate of 0.98% [17]. However, in our study, no cases of secondary phimosis were observed. We believe that the elliptical shape of the Alisklamp’s tip, as opposed to a completely round shape, reduces the incidence of secondary phimosis. The elliptical cut provides a wider diameter, which may help prevent this complication.

To the best of our knowledge, this study is the first randomized controlled trial that compares the dorsal slit technique and Alisklamp technique for male circumcision. Despite the rigorous design and execution of this multicentric randomized controlled trial, several limitations should be acknowledged. The study primarily included patients under 2 years old and a small number over 6 years old, which may not fully represent the outcomes in older children and adolescents. This limits the generalizability of our findings to all age groups. Although our sample size adhered to the guidelines, a larger cohort may have provided more robust data, particularly for rare complications. The follow-up period of one month may not capture all late postoperative complications, such as delayed infections or long-term cosmetic outcomes. The study was conducted in a specific geographic region, which may influence the generalizability of the results due to cultural and regional variations in surgical practices and patient care. Pain assessment was conducted using the FLACC scale, which is subjective and may not fully capture the pain experiences of all patients. Another limitation of our study is that we did not assess parental satisfaction. Future studies should address these limitations by including a more diverse age range, larger sample sizes, longer follow-up periods, and multi-regional data to enhance the generalizability and robustness of the findings. Additionally, standardized postoperative care protocols across all centers would help minimize bias and variability.

## 5. Conclusions

The Alisklamp technique demonstrated several advantages over the conventional dorsal slit technique for male circumcision. The Alisklamp method significantly reduced operation time and the rate and severity of penile edema. Pain scores were lower in the Alisklamp group at 10 min and 1 h post operation. Additionally, the incidence of skin tunnel formation was significantly lower in the Alisklamp group, leading to a more cosmetically appealing outcome.

The Alisklamp technique is recommended as the preferred method for circumcision because it minimizes complications, shortens the procedure time, and is easy to apply.

## Figures and Tables

**Table 1:** FLACC score.

Behaviour	0	1	2
Face	No particular expression or smile	Occasional grimace or frown, withdrawn, disinterested	Frequent to constant quivering chin, clenched jow
Legs	Normal position or relaxed	Uneasy, restless, tense	Kicking or legs drawn up
Activity	Lying quietly, normal position, moves easily	Squirming, shifting, back and forth, tense	Arched, rigid or jerking
Cry	No cry (awake or asleep)	Moans or whimpers; occasional complaint	Crying steadily, screams, sobs, frequent complaints
Consolability	Content, relaxed	Reassured by touching, hugging or being talked to, distractible	Difficult to console or comfort

Total score between 0–10. (0 = relaxed and comfortable); (1–3 = mild discomfort); (4–6 = moderate pain); (7–10 = severe discomfort/pain).

**Table 2:** Comparison of demographics.

	Group AK (n = 84)	Group DS (n = 82)	*p*-Value
Severe rotational anomaly	0 (0%)	1 (1%)	0.316
Meatal stenosis	0 (0%)	1 (1%)	0.316
Preoperative UTI	4 (5%)	4 (5%)	1.000
Preoperative phimosis	5 (6%)	4 (5%)	0.732
Circumcision reason			1.000
UTI	4 (5%)	4 (5%)	
Religion reasons	80 (95%)	78 (95%)	

UTI: Urinary tract infections.

**Table 3:** Comparison of outcomes.

	Group AK (n = 84)	Group DS (n = 82)	*p*-Value
Nausea	3 (3.6%)	6 (7.1%)	0.304
Vomiting	2 (2.4%)	4 (4.8%)	0.406
Penil oedema	2 (2.4%)	16 (19%)	<0.001
Oedema degree			0.019
Mild	2 (100%)	2 (12.5%)	
Moderate	0 (0%)	7 (43.8%)	
Severe	0 (0%)	7 (43.8%)	
Postoperative bleeding	1 (1.2%)	4 (4.8%)	0.173
Necrosis	0 (0%)	0 (0%)	NA
Local infection	1 (1.2%)	1 (1.2%)	1.000
Wound dehiscence	0 (0%)	1 (1.2%)	0.316
Wound gaping	7 (8.3%)	1 (1.2%)	0.030
Skin tunnel	0 (0%)	8 (9.5%)	0.004
Chordee after procedure	0 (0%)	0 (0%)	NA
Rotational anomaly after procedure	0 (0%)	0 (0%)	NA
Scondary phimosis	0 (0%)	0 (0%)	NA
Pain reliever at home > 2 times	16 (19%)	13 (15.5%)	0.540

NA: Not applicable.

## Data Availability

The datasets generated during and/or analyzed during the current study are available from the corresponding author on reasonable request.

## References

[1] (2012). American Academy of Pediatrics Task Force on Circumcision Male Circumcision. Pediatrics.

[2] Prabhakaran S., Ljuhar D., Coleman R., Nataraja R.M. (2018). Circumcision in the Paediatric Patient: A Review of Indications, Technique and Complications. J. Paediatr. Child Health.

[3] Сагір С., Азізoглу М., Ергюн М. (2023). Fewer Knots in Circumcision Are Associated with Less Postoperative Pain: A Retrospective Comparative Study. Неoнатoлoгія Хірургія Та Перинатальна Медицина.

[4] Musau P., Demirelli M., Muraguri N., Ndwiga F., Wainaina D., Ali N.A. (2011). The Safety Profile and Acceptability of a Disposable Male Circumcision Device in Kenyan Men Undergoing Voluntary Medical Male Circumcision. J. Urol..

[5] Hassan Y., Rasool H., Rather A.A., Ahmad Y., Rasool I. (2022). Religious Circumcision (Khatna) and Circumcision Mishaps in Kashmiri Children. Afr. J. Paediatr. Surg. AJPS.

[6] Toprak H., Kandemir E. (2024). Comparison of the Effects of Ring Block and Dorsal Penile Nerve Block on Parental Satisfaction for Circumcision Operation in Children: Randomized Controlled Trial. Pediatr. Surg. Int..

[7] Akman M. (2021). What is world pediatric surgeons’ opinion on EMLA® cream induced local anaesthesia in circumcision?. Çoc. Cer. Derg..

[8] Demir M., Eren H. (2020). Does the use of diapers have an effect on complications of circumcision?. Çoc. Cer. Derg..

[9] Warees W.M., Anand S., Leslie S.W., Rodriguez A.M. (2024). Circumcision. StatPearls.

[10] Obiero W., Young M.R., Bailey R.C. (2013). The PrePex Device Is Unlikely to Achieve Cost-Savings Compared to the Forceps-Guided Method in Male Circumcision Programs in Sub-Saharan Africa. PLoS ONE.

[11] Rao J.-M., Huang H., Chen T., Yang C.-G., Pan C.-Z., Deng G.-C., Shen L.-J., Qian X.-H., Peng M.-K., Zhou H.-D. (2020). Modified Circumcision Using the Disposable Circumcision Suture Device in Children: A Randomized Controlled Trial. Urology.

[12] Su Q., Gao S., Chen J., Lu C., Mao W., Wu X., Zhang L., Zuo L. (2020). A Comparative Study on the Clinical Efficacy of Modified Circumcision and Two Other Types of Circumcision. Urol. J..

[13] Feldblum P., Martinson N., Bvulani B., Taruberekera N., Mahomed M., Chintu N., Milovanovic M., Hart C., Billy S., Necochea E. (2016). Safety and Efficacy of the PrePex Male Circumcision Device: Results From Pilot Implementation Studies in Mozambique, South Africa, and Zambia. J. Acquir. Immune Defic. Syndr. 1999.

[14] Al Hussein Alawamlh O., Kim S.J., Li P.S., Lee R.K. (2018). Novel Devices for Adolescent and Adult Male Circumcision. Eur. Urol. Focus.

[15] Essa M. (2023). Safety, Acceptability, and Feasibility of Male Circumcision Using the Alisklamp Device. J. Pediatr. Urol..

[16] Alsowayan O.S., Al Zahrani A.M., Basalelah J.H., Al Madi M.K., Al Humam A.A., Al Otaibi A.N., AlKhamis A.A., Fadaak K.H., Al Suhaibani S.S., El Darawany H.M. (2024). A Prospective Randomized Controlled Trial Measuring Satisfaction and Parents Stress after Gomco and Plastibell Infant Circumcision. Pediatr. Surg. Int..

[17] Süzen A., Karakuş S.C., Ertürk N. (2021). Circumcision with Plastic Alisclamp Technique in 4733 Boys: Our Experiences to Reduce Complications. Turk. J. Med. Sci..

[18] Aydoğdu B., Azizoğlu M., Okur M.H. (2022). Social and Psychological Effects of Circumcision: A Narrative Review. J. Appl. Nurs. Health.

[19] Naja Z., Al-Tannir M.A., Faysal W., Daoud N., Ziade F., El-Rajab M. (2011). A Comparison of Pudendal Block vs Dorsal Penile Nerve Block for Circumcision in Children: A Randomised Controlled Trial. Anaesthesia.

[20] Osorio W., Ceballos C., Moyano J. (2022). Effectiveness of acute post-operative pain management by the acute pain service. Cir. Cir..

[21] Willis M.H., Merkel S.I., Voepel-Lewis T., Malviya S. (2003). FLACC Behavioral Pain Assessment Scale: A Comparison with the Child’s Self-Report. Pediatr. Nurs..

[22] Aldemir M., Çakan M., Burgu B. (2008). Circumcision with a New Disposable Clamp: Is It Really Easier and More Reliable?. Int. Urol. Nephrol..

[23] Senel F.M., Misirlioglu F. (2012). 1588 Comparison of Circumcisions Performed with Plastic Clamp and Conventional Dissection Technique. Arch. Dis. Child..

[24] Senel F.M., Demirelli M., Oztek S. (2010). Minimally Invasive Circumcision with a Novel Plastic Clamp Technique: A Review of 7500 Cases. Pediatr. Surg. Int..

[25] Karadag M.A., Cecen K., Demir A., Kivrak Y., Bagcioglu M., Kocaaslan R., Ari M., Altunrende F. (2015). SmartClamp Circumcision versus Conventional Dissection Technique in Terms of Parental Anxiety and Outcomes: A Prospective Clinical Study. Can. Urol. Assoc. J. J. Assoc. Urol. Can..

[26] Bawazir O.A., Banaja A.M. (2020). Sutureless versus Interrupted Sutures Techniques for Neonatal Circumcision; a Randomized Clinical Trial. J. Pediatr. Urol..

